# Latent profile analysis of family and school supports among Chinese adolescents in stepfamilies

**DOI:** 10.1111/famp.13086

**Published:** 2024-11-28

**Authors:** Yushan Zhao, Todd M. Jensen, Ashley Munger

**Affiliations:** ^1^ Human Development, Department of Human Ecology University of California Davis California USA; ^2^ Human Development and Family Science, School of Education University of North Carolina Chapel Hill North Carolina USA; ^3^ Department of Child and Family Studies California State University Los Angeles California USA

**Keywords:** adolescents, China, family, school, stepfamily, well‐being

## Abstract

Divorce and remarriage rates have increased dramatically in China, and more children live in stepfamilies. There remain valuable opportunities to understand the various family and school assets that support the well‐being of Chinese youth amid family structural transitions, such as the transition to stepfamily life. Using latent profile analysis, the current study seeks to identify patterns of youth support using seven family‐related variables and two school‐related variables as indicators among a sample of Chinese youth (*N* = 269; $M_{\mathrm{age}}$ = 14 years; 129 females and 117 males) residing with a parent and stepparent. Four profiles were identified: low support, academic focus/low support, moderate support, and high support. Results further demonstrated that youth in the moderate support profile had significantly better well‐being outcomes compared to youth in the low support or academic focus/low support profiles; demographic characteristics such as low SES families and parents with lower education backgrounds were associated with the low support profile; and stepfamilies with stepfathers were overrepresented in the moderate support profile, whereas stepfamilies with stepmothers were overrepresented in the low support and academic focus/low support profiles. These findings can inform the development of interventions intended to bolster the well‐being of Chinese adolescents in stepfamilies.

As Chinese divorce and remarriage rates have increased, more children live in diverse family structures, including stepfamilies—a family structure in which one or both of the parents have a child or children from a previous couple relationship (Chen et al., 2021; Ganong & Coleman, 2018). Mo (2017) found that the crude divorce rate, which is determined by the ratio of divorces to every 1000 individuals in the population, increased 178% from 1996 to 2013. Meanwhile, the refined divorce rate, which is calculated by tracking divorces among every 1000 women in marriage, rose by 211%. Likewise, the remarriage rate has also increased, resulting in a 50% increase in the percentage of Chinese children living in stepfamilies from 2010 to 2014 (Zhang, 2017). Research about Chinese stepchildren largely uses a deficit‐comparison approach, comparing the well‐being of children from stepfamilies to children from two‐parent, biologically connected families (Fung, 2021; Lan & Sun, 2022; Zhang, 2020).

Findings generally indicate children from stepfamilies disproportionately experience negative outcomes, such as lower academic performances, greater depression and anxiety, and greater externalizing behavioral problems (Fung, 2021; Lan & Sun, 2022; Zhang, 2020). As suggested by stepfamily research in contemporary Western countries—a deficit‐comparison approach to research in this area fails to provide practical guidance on what strategies help stepchildren function well in the family contexts they inhabit (Ganong et al., 2022), a new approach should also be adopted for studying stepfamily in the Chinese cultural context.

Researchers have identified protective factors within family and school settings in the Western context that can promote youth well‐being in varying family arrangements. Protective factors such as positive school climate (O'Malley et al., 2015), high‐quality parent–child relationships (Jensen, 2019, 2022), parental involvement (Ivanova & Kalmijn, 2020), parental monitoring in the usage of social media (Beckmeyer et al., 2020), family routines like having dinner together (Beckmeyer et al., 2020), and parent–child communication (Jensen, 2019) can benefit children within stepfamilies in terms of social–emotional development, behavioral development, and academic achievement. There is a dearth of such research focused on Chinese youth residing in stepfamilies and the family‐ and school‐related factors that cultivate their well‐being.

This cross‐sectional study aims to examine patterns of family‐ and school‐related support experienced by Chinese adolescents in stepfamilies. We also assess associations between these patterns and indicators of youth well‐being and sociodemographic characteristics. This study, to our knowledge, is the first to use a strengths‐based approach within the Chinese context to identify family and school supports that promote the well‐being of Chinese adolescents in stepfamilies. To begin, we describe trends associated with divorce and stepfamily formation in the Chinese context. Next, we articulate our critique of the deficit‐comparison approach and highlight a strength‐based approach as an optimal strategy for studying the well‐being of Chinese youth residing in stepfamilies. We then present our specific study aims.

## Divorce and stepfamily formation in Chinese context

Adjusting to a stepfamily is difficult, as it can involve many transitions (Lam, 2006), including the initial divorce, altered living and education arrangements, the formation of new parental relationships, remarriage, and merging households. While children experiencing transitions may develop coping strategies, too many transitions can overwhelm them, with mental health effects that can even last into adulthood (Shafer et al., 2017).

Complicated personal and family dynamics may interfere with adult figures' capacities to support children through these transitions (Ganong & Sanner, 2023). Unlike biologically connected two‐parent families, which have more time to develop different roles, stepfamily members are often thrust into predefined roles (e.g., a stepmother may become a mom without experiencing pregnancy and early stages of the child‐rearing) and don't have enough time to set expectations and boundaries for these roles. Children in stepfamilies who do not receive the support they need during these transitions are vulnerable to adjustment problems, including internalizing and externalizing issues (Jensen et al., 2018). Traditional Chinese culture adds to these transitions and dynamics and makes things more complicated. Traditional Chinese culture embraces Confucianism, which highly emphasizes the importance of family harmony and cohesion (Zhang, 2009). Divorce is seen as antithetical to these Chinese core values, and stepfamilies are considered “broken” or “dysfunctional” (Chen et al., 2021; Lam, 2006). Individuals within stepfamilies tend to hide their marital status, experience isolation, and lack community support because they are marginalized (Lam, 2006; Lam‐Chan, 1999). Moreover, traditional Chinese culture proposes “Ren,” which emphasizes tolerance as a way to deal with conflict and discourages open communication (Huang, 2000). In the stepfamily context, parents may not provide opportunities for stepchildren to speak up about their needs and confusion. Unsolved and hidden problems make stepchildren more vulnerable to negative outcomes such as mental health problems as they experience the transition to stepfamily life. Therefore, as more children experience parental divorce and live in stepfamilies, it is important to continually study the experiences of stepchildren in stepfamilies and identify key supports that cultivate their well‐being in the Chinese context.

## Moving away from a deficit‐comparison approach

Using the deficit‐comparison approach, many past studies contrast levels of individual well‐being across diverse family structures, often treating married, two‐parent, biologically connected families as the standard of comparison (Jensen & Sanner, 2021). Studies tend to reveal that children living in diverse family structures report relatively worse average scores on various indicators of well‐being compared to their counterparts in married, two‐parent, biologically connected families (Fung, 2021; Lan & Sun, 2022; Zhang, 2020).

The deficit‐comparison approach points out the disadvantages faced by diverse families but often fails to answer the question of what works well within diverse families, thus limiting the practical applicability of study findings (Ganong et al., 2022). The reason why individuals, especially children in stepfamilies, face unique challenges is because stepfamily is a more complicated family structure (Papernow, 2018). Common stepfamily challenges include but are not limited to (1) the complexities arising from changes in family structure, which necessitate adjustments in family processes (Hetherington et al., 1998); (2) the loss of a biological parent (i.e., a parent moving out of children's primary residence; Afifi & Keith, 2004); (3) stepparent–child conflict (Ganong & Coleman, 2017); (4) unclarity or ambiguity about optimal stepparent roles and functions (Cartwright, 2012; Jensen, 2021); (5) loyalty issues (e.g., children worrying that efforts to connect with a new stepparent could disrespect another parent; Papernow, 2018); (6) policy structures are geared towards two‐parent, non‐blended families and may not meet the specific needs of diverse families (Liu, 2018).

While these challenges may be faced by stepfamilies across cultures, the nature and severity of the challenges may vary. Relatively little research has analyzed stepfamily functioning across cultures. One study found that in collectivist cultures such as Israel, stepfamilies are more likely to perceive their family structure as similar to that of biologically connected, two‐parent families, as opposed to stepfamilies from individualist cultures like the US (Berger, 2000). This lack of differentiation could lead stepfamilies in collectivist cultures to unrealistic expectations and adherence to the traditional two‐parent family model as a standard for their own family dynamics. They also found that stepfamilies in collectivist cultures, Israel, for example, were less likely to seek support services, such as marriage therapy, compared to their counterparts in the individualist US culture. This disparity is often due to the stigma associated with mental health issues prevalent in many cultures (Berger, 2000; Xu et al., 2018). Although the study by Berger (2000) is limited to the US and Israel, it is plausible that stepfamilies in China, which also possesses a collectivist culture, may face similar challenges. Additionally, stepfamilies from cultural backgrounds that emphasize familism (e.g., Latino culture) are more likely to experience stigma regarding their family structure (Reck et al., 2012). This stigma is reinforced by the deficit‐comparison approach, which is particularly evident in Chinese culture, which values family harmony and views divorce and remarriage as norm violations (Lam‐Chan, 1999).

## A strengths‐based approach

Western researchers increasingly have moved away from a deficit‐comparison approach towards a strength‐based or normative‐adaptive approach, which prioritizes efforts to identify assets that promote individuals' well‐being within the family context they inhabit. In the context of stepfamilies, the relationships stepchildren have with different parental figures (i.e., non‐resident or resident biological parent, stepparent) are important to their well‐being. For example, Jensen et al. (2018) found that the relationship between biological parents and their children directly affects the mental health and behavior of young adolescents. However, the relationship between stepparents and stepchildren can have a more enduring influence on stepchildren's overall well‐being. A stepparent's warm and caring attitude towards stepchildren not only greatly benefits the stepchildren but also positively impacts the family climate (Jensen, 2022).

Parental involvement, monitoring, and communication have also been shown to benefit stepchildren's development. For example, stepchildren were found to experience fewer behavioral problems, lower depressive symptoms, and develop more positive relationships with their stepfathers when their stepfathers engaged more with them socially and academically (Jensen et al., 2018; Jensen & Pace, 2016; Yuan & Hamilton, 2006). Additionally, Beckmeyer et al. (2020) found that parental social media monitoring, family routines (i.e., having family meals), and communication about children's friends are positively associated with child well‐being in diverse family structures.

Outside of the family, school‐related factors have been found to buffer the negative impact of family stressors (including structural transitions) on child outcomes (O'Malley et al., 2015). Lan and Mastrotheodoros (2022) indicated that nurturing and encouraging teaching styles can buffer the negative impact of divorce on Chinese adolescents' mental health and behavioral problems. Positive school experiences can promote academic success, particularly among children living in stepfamilies and single families (Rodgers & Rose, 2001). In terms of the unstable family dynamics (e.g., new non‐biological parental figures, unclear roles) common to stepfamilies, school may provide a more stable and predictable environment that benefits child outcomes (Rodgers & Rose, 2001). A supportive and caring teacher can help buffer emotional stress among stepchildren whose families may struggle to fulfill their roles in providing support due to role ambiguity and complicated family dynamics (Jensen, 2020, 2021; Rodgers & Rose, 2001).

## Theoretical framework

Our analysis is informed by two theoretical frameworks. From a Family Systems perspective (Adamsons et al., 2022), stepfamilies may have unique and complex experiences that shape family relationships and functioning. First, the feedback from the environment may differ from non‐blended families, particularly when there is a stigma towards divorce and remarriage within the broader cultural context. Negative feedback may exacerbate stress and impact family dynamics. Second, because stepfamily formation differs from biologically connected, two‐parent families, stepfamilies may face challenges merging previously formed systems and developing and negotiating new rules, boundaries, expectations, and patterns of interaction. Additionally, families vary in the resources available to manage attendant conflict and stress associated with stigma or transitions. Families that experience greater stigma from their environment; that have ambiguous or strained rules, norms, and boundaries; or that face significant resource constraints to address internal and external challenges may experience poorer outcomes for individual family members.

Chinese cultural elements further complicate these family dynamics. Culturally, divorce and remarriage are stigmatized (Lam‐Chan, 1999). Filial piety, a central cultural value that expects young people to show respect and obedience to their elders, may influence the enforcement of family rules within these units. For example, parental monitoring—a method to enforce family rules related to childcare—typically has a more positive impact on parent–child relationships among adolescents who strongly adhere to filial piety (Wong et al., 2010). To understand what fosters positive child outcomes in Chinese stepfamilies, it is crucial to explore the intersection of complex family dynamics and Chinese cultural values.

Second, from a stress and support perspective, adolescents are at an increased risk for mental health problems if they live in environments with limited support and lack constructive engagement (Sheeber et al., 2001). Thus, understanding the role of family and school support becomes crucial in promoting the well‐being of adolescents, particularly in the context of stepfamily formation.

## The present study

The current study aims to identify patterns of family and school supports experienced by Chinese adolescents in stepfamilies using a person‐centered approach. Family‐structure researchers have commonly used a variable‐centered approach to assess associations between family structure and child well‐being, often comparing children from stepfamilies to those from two‐parent, biologically connected families (Fung, 2021; Zhang, 2017). We deviate from this approach by leveraging person‐centered analyses intended to identify meaningful patterns of family and school supports experienced by Chinese youth in stepfamilies. That is, the current study seeks to identify constellations of factors that might cultivate well‐being among these youth. Such an approach is particularly useful given the scant research on protective factors for Chinese children from stepfamilies.

Our specific research questions are as follows: (1) To what extent are there distinct patterns of family and school supports among Chinese adolescents residing in stepfamilies? (2) To what extent are varying patterns of family and school supports associated with the well‐being of Chinese adolescents residing in stepfamilies, specifically academic achievement, self‐efficacy, delinquent behaviors, and depression? (3) To what extent are varying patterns of family and school supports associated with sociodemographic factors (i.e., gender, age, number of siblings, SES, father and mother's educational background, stepfamily types)?

## METHOD

### Participants

Participants (i.e., adolescent respondents) for the current study were recruited from the Chinese Education Panel Survey (CEPS), a nationally representative longitudinal study focusing on the familial and educational experiences of adolescents in mainland China (National Survey Research Center, 2017). The IRB at Renmin University of China approved data collection. The baseline survey (wave 1) was administered to seventh and eighth graders throughout the academic years of 2013 to 2014 using a multi‐staged probability proportional to size sampling approach. The data collection for the CEPS project included several procedures. They first used two characteristics—educational attainment and internal migration rate—to categorize all districts in China and then randomly select 28 districts, either from urban or rural areas. Second, they used other criteria—data of enrollment and type of school—to randomly select four schools from each selected 28 districts, resulting in 112 schools. Third, they selected four classes from the above‐selected schools, resulting in 448 classrooms in total. This study used the follow‐up data (wave 2) collected between 2014 and 2015, which incorporated information on parents' marital status that was absent in the Wave 1 data. A total of 269 Chinese adolescents in stepfamilies (129 females and 117 males) aged 12 to 17 years old ($M_{\mathrm{age}}$ = 14 years) were included in this study. Three types of stepfamilies were identified: those living with a stepfather (47%), stepmother (49%), or both (4%). The majority of the participants were Han (88%) and the remaining were other ethnic minorities (12%). About 37% of the participants did not have half or full siblings, whereas 63% of the participants did. However, the sibling item did not specify the number of half siblings participants have. About 66% of the participants did not live with grandparents, whereas 34% did. Approximately 19.9% of the participants indicated living in a very poor or somewhat poor family, 71.5% living in a moderate, and 8.6% living in a rich or very rich family.

### Procedure

The surveys were written in Chinese and developed by the CEPS scholars; we obtained the English version of the survey from the CEPS website (Chinese Education Panel Survey, n.d.). Several scales were included to measure adolescents' family and school experiences. Most of the scales were pulled from the Wave 2 student survey. Because the variables age, gender, and parental educational backgrounds were missing in the wave 2 student survey, we obtained this information from the wave 1 student survey and the wave 2 parent survey. We determined adolescents' ages during wave 2 by adding 1 year to the age variable during wave 1 to account for the year differences between the two waves. Father‐child closeness, mother–child closeness, parental academic involvement, and depression scales have been used in previous work with Chinese populations; however, the remaining scales have not been specifically validated with this population (Huo et al., 2020; Wang & Cai, 2017; Zheng et al., 2020).

### Measures of demographic variables

We measured six demographic variables: gender (“What is your gender?” coded as 0 = *female*, 1 = *male*), age (“How old are you?” with participants specifying their age), socioeconomic status, number of siblings, and parents' educational background.

#### Socioeconomic status (SES)

The students’ family SES was measured by a single question, “How's the financial condition of your family?” Participants rated their answers on a Likert scale: 1 (*very poor*), 2 (*somewhat poor*), 3 (*moderate*), 4 (*somewhat rich*), and 5 (*very rich*).

#### Number of siblings

Participants reported the number of full or half siblings they currently have by specifying the count for each sibling category: elder brother(s), younger brother(s), elder sister(s), and younger sister(s). Responses were recorded as numerical values, with participants instructed to enter ‘0’ if they did not have any siblings in a specific category. This question asked about the number of full or half siblings but did not allow us to distinguish between them, leaving out specific details on the number of half siblings versus full siblings.

Parents' educational background. Parents' education backgrounds are measured by a single question, “What is the highest education level you have completed?” Answers are rated from 1 (*None*), 2 (*Finished elementary school*), 3 (*Junior high school degree*), 4 (*Technical secondary school or technical school degree*), 5 (*Vocational high school degree*), 6 (*Senior high school degree*), 7 (*Junior college degree*), 8 (*Bachelor degree*), and 9 (*Master degree or higher*). A higher score on this question indicates a higher level of parental educational background.

### Measures of indicators for latent profile analysis

#### Father–child closeness

Adolescents were asked how they would describe their relationship with their father, with response options ranging from 1 (*not close*) to 3 (*very close*).

#### Mother–child closeness

Adolescents were asked how they would describe their relationship with their mother, with response options ranging from 1 (*not close*) to 3 (*very close*).

#### Parental academic involvement

Adolescents answered two questions to indicate their parents' frequency of involvement in their schoolwork, with response options ranging from 1 (*never*) to 4 (*almost every day*). The specific questions were as follows: “How often did your parents check up on your homework last week?” and “How often did your parents instruct your homework last week?” A composite score was calculated by averaging the scores of these two items. The two items yielded acceptable levels of internal consistency reliability in the sample (*α* = 0.71).

#### Parental social involvement

Adolescents answered three questions to indicate the frequency of their parents' involvement in their social life, with response options ranging from 1 (*never*) to 6 (*more than once a week*). The specific questions were as follows: “How often do you have dinner with your parents?”, “How often do you watch movies, shows, sports games, etc. with your parents? and “How often do you visit museums, zoos, science museums, etc. with your parents?” A composite score was calculated by averaging the scores of these three items. The three items yielded acceptable levels of internal consistency reliability in the sample (*α* = 0.70).

#### Parental monitoring

Adolescents answered six questions to describe the level of monitoring from their parents in different activities, with response options ranging from 1 (*they don't care*) to 3 (*they are very strict about it*). The specific questions were as follows: “Do your parents care and are they strict with you about the following? (1) Your homework and examination; (2) Your behavior at school; (3) Whom you make friends with; (4) Your dress style; (5) Time you spend on the Internet; and (6) Time you spend on watching TV.” A composite score was calculated by averaging these six items. The six items yielded acceptable levels of internal consistency reliability in the sample (*α* = 0.75).

#### Father‐child communication

Adolescents answered four questions about the frequency of communication they have with their father, with response options ranging from 1 (*never*) to 3 (*often*). The specific questions were as follows: “How often does your father discuss the following with you? (1) Things happened at school; (2) The relationship between you and your friends; (3) The relationship between you and your teachers; and (4) Your worries and troubles.” A composite score was created by taking the average of these four items. The four items yielded acceptable levels of internal consistency reliability in the sample (*α* = 0.83).

#### Mother–child communication

Adolescents were asked the same four questions related to communication with their mothers. A composite score was created by taking the average of these four items, and the items yielded acceptable levels of internal consistency reliability in the sample (*α* = 0.90).

#### Teacher attention

Adolescents reported their level of agreement (1 = *strongly disagree*, 4 = *strongly agree*) with nine statements describing how much attention they received from three types of teachers: math, Chinese, and English. The questions covered three specific aspects for each teacher: “My [teacher] (1) always pays attention to me; (2) always asks me to answer questions in class; and (3) always praises me.” A composite score was created by taking the average of these nine items, with a higher score indicating more teacher attention. The nine items yielded acceptable levels of internal consistency reliability in the sample (*α* = 0.90).

#### School experience

Adolescents reported their level of agreement (1 = *strongly disagree*, 4 = *strongly agree*) with 10 statements about their school experience. These statements were: (1) My parents always receive positive praise on me from my teacher; (2) My parents always receive criticism on me from my teacher; (3) My homeroom teacher always praises me; (4) My homeroom teacher always criticizes me; (5) Most of my classmates are nice to me; (6) My class is in a good atmosphere; (7) I often take part in school/class activities; (8) I feel close to people in this school; (9) I feel bored in this school; and (10) I hope that I could transfer to another school. Items two, four, nine, and 10 were reverse‐coded so the interpretation could be consistent across the scale. A composite score was created by taking the average of these 10 items, with a higher score indicating a more positive school experience. The 10 items yielded acceptable levels of internal consistency reliability in the sample (*α* = 0.71).

### Measures of youth well‐being

#### Self‐efficacy

Self‐efficacy was measured with the following four items: (1) I would try my best to go to school even if I was not feeling very well or I had other reasons to stay at home; (2) I would try my best to finish even the homework I dislike; (3) I would try my best to finish my homework, even if it would take me quite a long time; and (4) I would persist in my interests and hobbies. Response options ranged from 1 (*strongly disagree*) to 4 (*strongly agree*). A composite score was created by taking the average of these four items, with a higher score indicating more self‐efficacy. The four items yielded acceptable levels of internal consistency reliability in the sample (*α* = 0.80).

#### Academic achievement

Adolescents' academic achievement was assessed based on three compulsory subjects: Chinese, English, and Math. We standardized these scores by dividing the obtained score by the highest possible score. For instance, if a student scored 100 on an exam with a maximum score of 120, their standardized score would be 100/120 = 0.83. After determining the standardized scores for each subject, we took an average of these standardized scores across the three subjects as an index of adolescents' academic achievement. This approach has been used in broader academic evaluations such as American College Testing (ACT; Bettinger et al., 2013). In the ACT, the average of scores from different subjects is calculated as a composite score, which universities in the United States use to set admission cutoffs.

#### Depression

Adolescents' depressive symptoms were measured by the adapted version of the Center for Epidemiologic Studies Depression Scale (CES‐D) using the following 10 items: “How often did you have the following feeling in the past seven days? (1) Feeling blue; (2) Too depressed to focus on anything; (3) Unhappy; (4) Not enjoying life; (5) Having no passion to do anything; (6) Sad, sorrowful; (7) Nervous; (8) Excessive worry; (9) Feeling something bad will happen; and (10) Too energetic to concentrate in class” (Radloff, 1977). This scale has been used successfully among Chinese adolescents (Huo et al., 2020). Response options ranged from 1 (*never*) to 5 (*always*). A composite score was created by taking the average of these 10 items, with a higher score indicating a higher level of depression. The 10 items yielded acceptable levels of internal consistency reliability in the sample (*α* = 0.93).

#### Delinquent behaviors

Participants' delinquent behaviors were measured with the following 10 items: “How often did you do the following things in the past year? (1) Cursing or saying swearwords; (2) Quarreling with others; (3) Having a physical fight with others; (4) Bullying the weak; (5) Having a violent temper; (6) Unable to concentrate on one thing; (7) Skipping classes, being absent, or truanting; (8) Copying homework from others, or cheating in exams; (9) Smoking, or drinking alcohol; and (10) Going to net bars or video arcade.” Response options ranged from 1 (*never*) to 5 (*always*). A composite score was created by taking the average of these 10 items, with a higher score indicating higher levels of delinquent behaviors. The 10 items yielded acceptable levels of internal consistency reliability in the sample (*α* = 0.86).

### Data analysis

Data preparation and management were conducted using R, and latent profile analysis (LPA) was conducted using Mplus 8.10. LPA is a person‐centered approach where all observed indicators of interest are continuous variables (Weller et al., 2020). LPA seeks to identify unobserved subgroups that possess similar patterns of responses across the observed indicators of interest (Weller et al., 2020).

The LPA was conducted following the steps outlined by Sinha et al. (2021) and Asparouhov and Muthén (2014). We first prepared standardized forms of our continuous measures of various family and school supports, which served as our focal observed indicators for the LPA. We then engaged in a profile enumeration process, whereby we leveraged several statistical indices to assess the relative fit of solutions with varying numbers of profiles specified. Focal statistical indices included the Akaike Information Criterion (AIC), Bayesian Information Criterion (BIC), adjusted BIC (aBIC), Vuong‐Lo–Mendell–Rubin likelihood ratio test (LRT with p‐value), Lo–Mendell–Rubin adjusted likelihood ratio test (aLRT with p‐value), bootstrap likelihood ratio test (BLRT with p‐value), mean posterior probability values, and entropy. Generally, lower AIC, BIC, and aBIC values signal better model fit; significant likelihood ratio tests also indicate that *k* number of profiles could be preferred over *k‐1* number of profiles (Nylund et al., 2007). We also examined the number of cases for each estimated profile, preferring that the smallest profile not possess fewer than 30 cases (representing our effort to avoid an over‐extracted profile solution; Weller et al., 2020). After identifying the optimal profile solution, we assessed associations between profile membership and covariates, specifically measures of youth well‐being (e.g., academic achievement) and sociodemographic characteristics (i.e., SES, stepfamily types) using the automated three‐step procedure in Mplus (Asparouhov & Muthén, 2014).

## RESULTS

### Profile enumeration

AIC and aBIC decreased as the profile number increased from one to six. The BIC also decreased from the one‐ to five‐profile solution and then increased slightly in the six‐profile solution. Despite the five‐profile solution having the lowest BIC, its smallest n was under 30, whereas the four‐profile solution's smallest n exceeded 30, suggesting the latter is the optimal choice per our pre‐specified criteria for evaluating solutions. Additionally, the four‐profile solution yielded mean posterior probabilities above 0.85 and an entropy value of 0.87. The five‐profile solution essentially partitioned the third profile, the moderate support profile, into two groups that were similar except for some minimal variation on academic involvement (one with moderately low levels and the other with just above moderately low levels). From a substantive perspective, the five‐profile solution did not add a profile with meaningful information beyond the four‐profile solution. Taken together, the four‐profile solution was selected as optimal. See Table 1 for more details about the profile enumeration process.

**TABLE 1:** Profile enumeration.

Profiles	AIC	BIC	aBIC	*p*‐values			Entropy	Smallest *n*	Mean posterior probabilities					
				LRT	aLRT	BS LRT			1	2	3	4	5	6
1	6766.94	6831.65	6774.57											
2	6466.14	6566.80	6478.02	0.00	0.00	0.00	0.81	103.00	0.94	0.94				
3	6377.09	6513.69	6393.20	0.02	0.02	0.00	0.85	50.00	0.93	0.93	0.94			
4	6334.41	6506.96	6354.76	0.48	0.48	0.00	0.87	32.00	0.95	0.94	0.86	0.94		
5	6280.66	6489.15	6305.26	0.44	0.44	0.00	0.89	21.00	0.92	0.92	0.98	0.92	0.96	
6	6264.53	6508.97	6293.36	0.85	0.85	0.05	0.87	21.00	0.94	0.82	0.92	0.84	0.97	0.97

### Optimal latent‐profile solution

**FIGURE 1:**
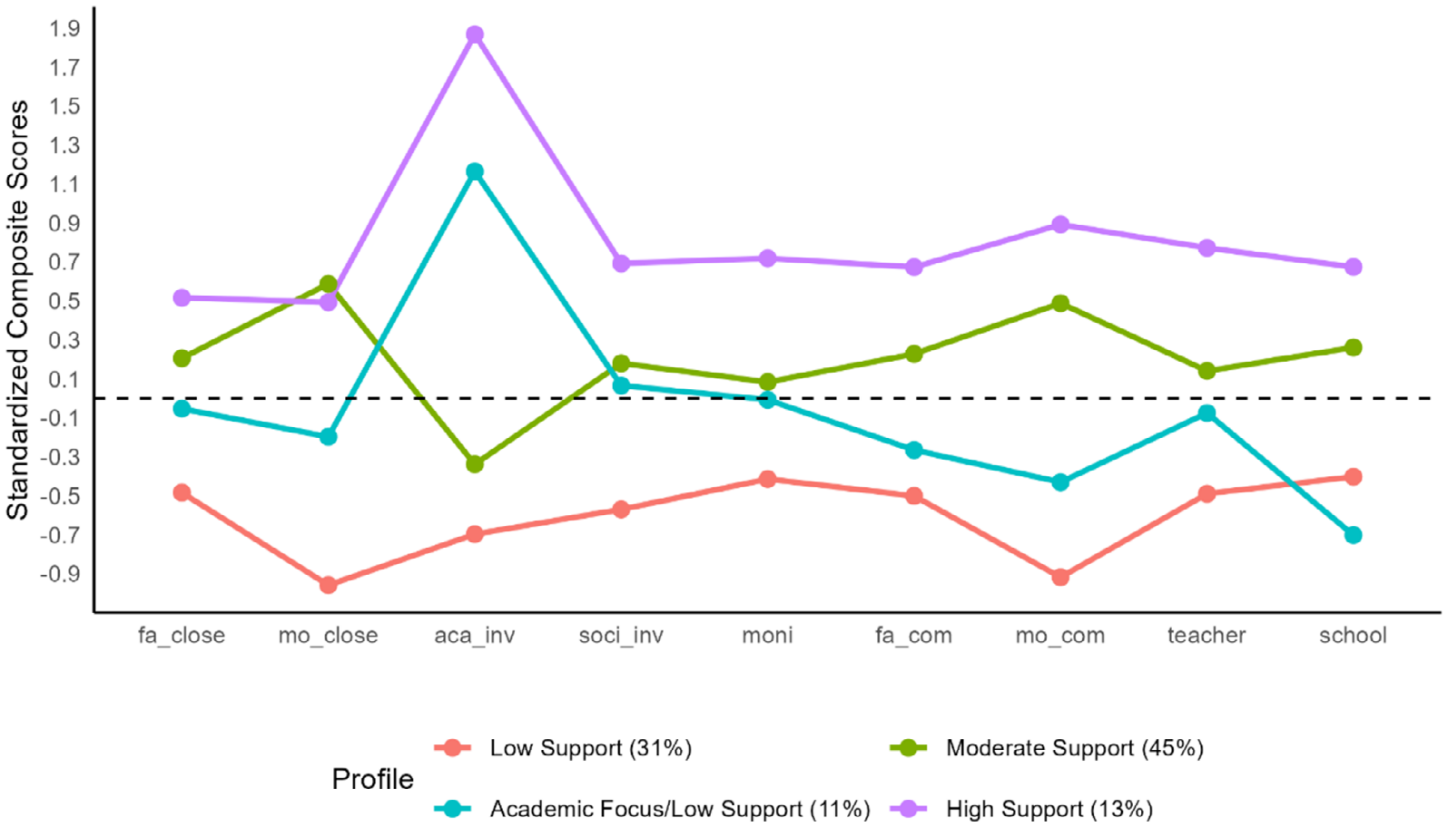
Visualization of the latent profile solution. aca_inv, parental academic achievement; f_close, father‐child closeness; f_com, father‐child communication; m_close, mother–child closeness; mo_com, mother–child communication; moni, parental monitoring; school, school experience; soc_iv, social involvement; teacher, teacher attention.

The four‐profile solution is visually presented in Figure 1. Profile 1 was labeled as low support (*n* = 84, 31% of the sample). Youth belonging to this profile displayed relatively low mean levels across all indicators (near or below 0.5 standard deviations below the sample mean). Profile 2 was labeled as an academic focus but low support (*n* = 31, 11% of the sample). Youth belonging to this group fell 1.2 standard deviations above the sample mean with respect to parental academic involvement, with the remaining family and school experience indicators falling below sample‐mean levels. Especially for school experience, this group of youth fell 0.7 standard deviations below the sample mean, representing the lowest average value for this indicator across all four profiles. Profile 3 was labeled as moderate support (*n* = 120, 45% of the sample). Youth belonging to the moderate support profile possessed high levels of mother–child closeness and mother–child communication (0.6 standard deviations above the sample mean). However, in terms of parental academic involvement, this group of youth possessed below‐average levels (0.3 standard deviations below the sample mean). Other family and school experiences for this group were near or above sample‐mean levels. Profile 4 was labeled as high support (*n* = 34, 13% of the sample). Youth belonging to the high support profile possessed notably high levels across all indicators—nearly two standard deviations above the sample mean in terms of parental academic involvement and more than 0.5 standard deviations above the sample mean across the other family and school experience variables.

### Profile differences

Table 2 shows the mean differences in youth well‐being across the four profiles. In terms of self‐efficacy and depression, youths in the low support profile displayed significantly lower self‐efficacy and showed increased symptoms of depression than youths in the moderate or high support profiles. Similarly, youths in the academic focus/low support groups exhibited higher levels of depression than youths in the moderate or high support profiles. Concerning academic achievement, youths in the moderate support profile performed significantly better than youths in the academic focus/low support or low support profile. Only one pair of differences was found regarding delinquent behavior: youths in the low support profile engaged in more delinquent behaviors than those in the moderate support profile. Rating the mean values of youth well‐being in each category, youths in the moderate support profile displayed the highest values in self‐efficacy and academic achievement and exhibited the lowest values in depression and delinquent behaviors. However, these values were not significantly different from youth in the high support profile.

**TABLE 2:** Latent profile differences.

Full sample (*N* = 269)		Low support (31%) (*n* = 84)		Academic focus/low support (11%) (*n* = 31)		Moderate support (45%) (*n* = 120)		High support (13%) (*n* = 34)		Profile differences, *p ≤ 0.05*
Covariate	M	M	SE	M	SE	M	SE	M	SE	
Youth well‐being										
Self‐efficacy	3.14	2.87	0.09	3.09	0.16	3.26	0.06	3.49	0.11	3 > 1; 4 > 1
Academic achievement	0.62	0.56	0.02	0.52	0.05	0.68	0.02	0.64	0.03	3 > 2; 3 > 1
Depression	2.36	2.54	0.11	2.91	0.26	2.15	0.08	2.16	0.16	2 > 3; 2 > 4; 1 > 3; 1 > 4
Delinquent behaviors	1.59	1.78	0.07	1.54	0.11	1.49	0.04	1.54	0.16	1 > 3
Demographic characteristics										
Gender	0.52	0.54	0.06	0.38	0.05	0.55	0.10	0.54	0.09	
Age	13.97	14.24	0.11	14.0	0.24	13.80	0.08	13.70	0.17	1 > 3; 1 > 4
Socioeconomic status	2.86	2.73	0.08	2.77	0.13	2.95	0.07	2.94	0.07	3 > 1
Number of siblings	0.83	1.09	0.12	0.60	0.14	0.73	0.08	0.74	0.12	1 > 2; 1 > 3; 1 > 4
Parental background										
Father's education	4.00	3.36	0.18	4.22	0.45	4.22	0.19	4.63	0.37	3 > 1; 4 > 1
Mother's education	3.89	3.22	0.18	4.07	0.40	4.18	0.19	4.32	0.33	3 > 1; 4 > 1
Stepfamily type										
Stepfather	0.49	0.32	0.06	0.27	0.09	0.64	0.05	0.60	0.09	3 > 2; 4 > 2; 3 > 1; 4 > 1
Stepmother	0.47	0.63	0.06	0.63	0.10	0.35	0.05	0.34	0.09	2 > 3; 1 > 3; 1 > 4
Stepfather and stepmother	0.04	0.05	0.03	0.12	0.15	0.01	0.01	0.05	0.09	

*Note*: Three‐step procedure was used to compute the means and estimate mean differences. This method accounts for classification uncertainty. For dummy variables (i.e., gender and stepfamily type), means stand for profile‐specific percentages.

Table 2 also shows the association between demographic variables (i.e., parental background) and profile membership. In terms of gender, there were no significant gender differences across profile membership. Youth in the low support profile were older and had more siblings compared with youth in the moderate and high support profiles. Youth in the moderate support profile reported higher SES compared to youth in the low support profile. Regarding parental background, the parents of youth in the low support profile were more likely to have lower educational backgrounds compared to youth in the moderate support or high support profiles.

For the stepfamily types, the predicted probability of students living with stepfathers is 32% for low support, 27% for academic focus but low support, 64% for moderate support, and 60% for high support. Youth living with stepfathers were predominantly represented in the moderate support profile. The predicted probability of students living with stepmothers was 63% for low support, 63% for academic focus but low support, 35% for moderate support, and 34% for high support. Youth living with stepmothers were predominantly represented in the low support profile and academic focus/low support profile.

## DISCUSSION

Past research has largely focused on outcome differences across children residing in various family structures in Western contexts (Jensen & Sanner, 2021; O'Malley et al., 2015). There remain important opportunities to identify factors that promote the well‐being of children residing in stepfamilies in Eastern contexts, including China. This study aimed to (1) identify distinct patterns of family and school supports experienced by Chinese adolescents residing in stepfamilies; (2) compare levels of well‐being across adolescents within each distinct pattern or profile of family and school supports; and (3) assess the extent to which profile membership was associated with sociodemographic factors. Our study showed that most stepchildren were in the moderate support profile (45%), and it is uncommon for stepchildren to experience high support in both family and school (13%).

What is clear is that stepchildren who experienced low supportive environments in both school and family displayed negative outcomes in almost every domain of their well‐being, which is supported by the stress and support perspective (Sheeber et al., 2001). Tailored interventions to bolster support within both family and school settings are most urgent for stepchildren in this group. For stepchildren in the academic focus/low support profile, Chinese stepparents or resident biological parents may involve themselves in the stepchildren's academic work as a means of support (Chen et al., 2021). This is because parents in stepfamilies might not know how to fulfill the stepparent role or assist their partner with this new role, given the ambiguity that can accompany the stepparent role (Jensen, 2021). However, for stepchildren who face many challenges, an excessive focus on academics without prioritizing supportive family dynamics, such as parental warmth, is likely to be suboptimal.

The low support profile and its associated negative outcomes may reflect an overall strain on resources (e.g., money and time) and high stress among some stepfamilies. Parents in a family with low SES may not have enough time and resources to invest sufficiently in every child. Thus, when more siblings exist in stepfamilies, stepparents tend to prioritize their younger biological children (Stewart, 2005). According to the family stress model, parents who face financial stress will be vulnerable to mental health problems, which will further influence their parenting capabilities (Conger et al., 2010). Moreover, parents in lower‐income families often live in rural communities with less convenient facilities compared to their higher‐income counterparts. This can make it challenging for them to bring their children to places like museums, zoos, and science centers, which may not be easily accessible or available to residents of rural areas and some small towns (Xu et al., 2022). Additionally, this result implied it is the moderate support, not the high support profile, that gives optimal outcomes, despite the lack of significant differences in their well‐being between these two profiles. This may be due to the small sample size of stepchildren in the high support profile (*n* = 34), which limits statistical power. It could be possible that high support is the most beneficial, but that cannot be determined given the sample size. Jensen (2019) found that levels of youth well‐being were near‐ or above average in several patterns of youth–stepparent interaction (academically oriented, casually connected, and versatile and involved). However, pathologizing the lack of high involvement within this family type ignores the challenges faced by stepparents within such a family structure. A moderately supportive family environment appears minimally sufficient for supporting stepchildren's well‐being (Jensen, 2019). Moreover, as the participants in this study are adolescents, providing moderate support (e.g., less monitoring) may align developmentally with their autonomy needs and facilitate their personal identity growth (Hughes et al., 2017). Notably, among the stepchildren in the moderate support profile, parental academic involvement is lower than in both the academic focus and high support profiles and is nearly as low as in the low support profile. This finding aligns with previous research indicating that what stepchildren need most is not academic discipline but social–emotional support—such as parent–child communication and social involvement, as examined in the past study (Ganong et al., 2022).

Lastly, stepchildren living with their stepfather were overrepresented in the moderate support profile, a profile that was linked to positive youth well‐being. In contrast, stepchildren living with stepmothers were overrepresented in the low support or academic focus/low support profile, two profiles that were linked to relatively lower levels of youth well‐being. This result aligned with past research in both the US and China indicating that stepchildren living with stepmothers display more negative outcomes compared with those living with stepfathers (Fine & Kurdek, 1992; Fung, 2021). From the perspectives of Chinese culture and family system theory, the stepfather‐biological mother family aligns more closely with the cultural values of “*Nan Zhu Wai*” (i.e., the husband should work outside) and “*Nv Zhu Nei*” (i.e., the wife should manage the household). In households with a stepfather, the biological mother naturally assumes the caregiving role, fulfilling the expectation of “*Nv Zhu Nei*.” Conversely, in households with stepmothers who are also expected to fulfill the role of “*Nv Zhu Nei*,” there may be struggles in adopting this role (Shapiro, 2014). One typical struggle faced by stepmothers is rejection from stepchildren due to loyalty issues—stepchildren may experience a loyalty bind towards their nonresident biological mother, making the parenting role especially difficult. A study on adult stepchildren found that the level of support stepmothers provide is mainly determined by the involvement of the biological mother (Van Houdt et al., 2020). Family systems theory also emphasizes the interaction among family subsystems (Cox & Paley, 2003). A stepmother's difficulty in assuming maternal roles may influence her marital relationship, which can, in turn, affect her relationship with her stepchildren and child outcomes. Indeed, research has found that both marital quality and the stepparent–child relationship are crucial for stepfamily functioning (Ganong et al., 2019). The significant barriers stepmothers face in performing maternal roles can impact broader contexts, including child outcomes, marital relationships, and overall family functioning. Challenges exist, yet stepmothers in China may not seek professional support for their maternal roles due to the stigma surrounding mental health services and the limited availability of such resources in the country (Xu et al., 2018). Without receiving positive “feedback” (e.g., therapy service) from their external environment and suffering from negative “feedback” (e.g., stigma), stepmothers and their families may be more susceptible to maladaptive dynamics, according to family systems theory. Taken together, stepmothers may want to fulfill their maternal roles but face significant challenges in doing so. Lacking a main caretaker, the biological mother, in this case, stepchildren might experience an overall less supportive family environment.

### Practical implications

There are several implications from the findings to support practice and future research. First, even moderate support may be sufficient to promote stepchildren's well‐being. While Chinese parents often place a strong emphasis on academic achievement, stepchildren may benefit more from moderate social–emotional support, such as regular communication and social involvement. Second, stepchildren in low‐support families may need support across numerous environments, including home and school. For example, the school can deliver training to teachers to understand stepchildren's family contexts, preventing perceptions of these children as disadvantaged (Claxton‐Oldfield & Voyer, 2001). Such efforts could contribute to the creation of supportive relationships and communities at school, which can help at‐risk children reduce risky behaviors and experience well‐being (Foster et al., 2017). Third, low‐support families may be especially vulnerable to resource constraints, which may undermine their ability to provide support. Intervention programs should support these stepfamilies by providing specific strategies and prevention techniques that strengthen family function and relationship quality (Michaels, 2006). For example, at the macro level, economic supports and policies could play a role in helping these families reach financial stability, which would lessen stress and support adaptive family processes. Additionally, at the interpersonal level, interventions concerning building supportive family relationships tailored to stepfamily strengths and challenges may be helpful. Fourth, this study does not aim to confirm the “wicked stepmother” stigma but recognizes the challenges stepmothers face, including societal stigma and a lack of role clarity. Interventions should aim to shift traditional gender norms and educate stepfamilies on the importance of shared housework and childcare (Shapiro, 2014). Support groups are also vital, offering stepmothers a community to share experiences and combat loneliness (Riness & Sailor, 2015), helping them navigate their roles more effectively.

### Limitations

This study did not evaluate the impact of aspects of Chinese culture (e.g., familism and avoidant communication style) on stepchildren's family and school experience, which can potentially impact stepchildren's well‐being (Huang, 2000; Lam, 2006). Additionally, the survey did not distinguish between non‐resident biological, resident biological, or stepparent figures, although past research suggests each parental figure influences stepchildren differently (Jensen & Harris, 2017). Furthermore, longitudinal studies are needed to better understand forms of support and their impacts over time. Family systems theory views the family as dynamic, not static, indicating stepchildren's family and school experiences likely evolve over time (Cox & Paley, 2003). Lastly, future research should incorporate in‐depth qualitative studies, or collect dyadic or triadic data, to provide Chinese practitioners and scholars with a deeper understanding of the complex family dynamics in stepfamilies. Limitations notwithstanding, this study addresses important gaps in the literature and offers a novel assessment of Chinese adolescents in stepfamilies regarding the various family and school assets that support their well‐being.
